# User-Centered Adaptation of an Existing Heart Failure Telemonitoring Program to Ensure Sustainability and Scalability: Qualitative Study

**DOI:** 10.2196/11466

**Published:** 2018-12-06

**Authors:** Patrick Ware, Heather J Ross, Joseph A Cafazzo, Audrey Laporte, Kayleigh Gordon, Emily Seto

**Affiliations:** 1 Institute of Health Policy, Management and Evaluation Dalla Lana School of Public Health University of Toronto Toronto, ON Canada; 2 Centre for Global eHealth Innovation Techna Institute University Health Network Toronto, ON Canada; 3 Ted Rogers Centre for Heart Research University Health Network Toronto, ON Canada; 4 Department of Medicine University of Toronto Toronto, ON Canada; 5 Peter Munk Cardiac Centre University Health Network Toronto, ON Canada; 6 Institute of Biomaterials and Biomedical Engineering University of Toronto Toronto, ON Canada; 7 Canadian Centre for Health Economics Toronto, ON Canada

**Keywords:** telemonitoring, mHealth, diffusion of innovation, heart failure

## Abstract

**Background:**

Telemonitoring interventions for the management of heart failure have seen limited adoption in Canadian health systems, but isolated examples of telemonitoring programs do exist. An example of such a program was launched in a specialty heart failure clinic in Toronto, Canada, and a recent implementation evaluation concluded that reducing the cost of delivering the program is necessary to ensure its sustainability and scalability.

**Objective:**

The objectives of this study were to (1) understand which components of the telemonitoring program could be modified to reduce costs and adapted to other contexts while maintaining program fidelity and (2) describe the changes made to the telemonitoring program to enable its sustainability within the initial implementation site and scalability to other health organizations.

**Methods:**

Semistructured interviews probed the experiences of patients (n=23) and clinicians (n=8) involved in the telemonitoring program to identify opportunities for cost reduction and resource optimization. Ideas for adapting the program were informed by the interview results and prioritized based on (1) potential impact for sustainability and scalability, (2) feasibility, and (3) perceived risks to negatively impacting the program’s ability to yield desired health outcomes.

**Results:**

A total of 5 themes representing opportunities for cost reduction were discussed, including (1) Bring Your Own Device (BYOD), (2) technical support, (3) clinician role, (4) duration of enrollment, and (5) intensity of monitoring. The hardware used for the telemonitoring system and the modalities of providing technical support were found to be highly adaptable, which supported the decision to implement a BYOD model, whereby patients used their own smartphone, weight scale, and blood pressure cuff. Changes also included the development of a website aimed at reducing the burden on a technical support telehealth analyst. In addition, the interviews suggested that although it is important to have a clinician who is part of a patient’s circle of care monitoring telemonitoring alerts, the skill level and experience were moderately adaptable. Thus, a registered nurse was determined to be more cost-effective and was hired to replace the existing nurse practitioners in the frontline management of telemonitoring alerts and take over the technical support role from a telehealth analyst.

**Conclusions:**

This study provides a user-centered example of how necessary cost-reduction actions can be taken to ensure the sustainability and scalability of telemonitoring programs. In addition, the findings offer insights into what components of a telemonitoring program can be safely adapted to ensure its integration in various clinical settings.

## Introduction

### Background

Meta-analyses have shown that telemonitoring for patients with heart failure (HF) can improve patients’ health outcomes and reduce health care utilization [1-4]. However, when one considers that HF directly impacts 1 million Canadians [5] and that in 2013 only 5000 patients across all disease types were enrolled in a telemonitoring program [6], it is clear that the diffusion of telemonitoring is lagging. This can partly be explained by higher than anticipated costs of implementing these programs and the lack of user input in the conception of such interventions [7]. In addition, although meta-analyses generally conclude positive outcomes, inconsistencies at the individual study level, particularly with respect to the economic impact, are difficult for stakeholders to ignore [8]. We have proposed in a previous work that this heterogeneity is caused by variances in the characteristics of (1) patients enrolled, (2) the intervention (eg, telemonitoring system used, clinician involvement, and supporting health services), and (3) fidelity with which the intervention is administered over time [8].

### Adaptability is Needed for Scalability

Although differences in the way telemonitoring interventions are delivered can lead to contradictory evidence, understanding these differences and how they might influence outcomes could hold one of the keys to scalability. This is because theories of diffusion of innovation have suggested that to be sustained and scaled, interventions must be able to adapt if they are to be embedded within local conditions [9,10]. This notion of adaptability is a prominent theme in studies of delivering digital health interventions at scale, which reinforce the view that a *one-size-fits-all* approach does not work [11]. The challenge is determining how to undertake necessary adaptations without compromising program fidelity [12].

A useful analogy used by theorists to discuss the notion of adaptability is the idea that health interventions have a *hard core* and a *soft periphery* [13-15]. The hard core represents the essence of an intervention, in other words, the central mechanism(s) for producing desired health outcomes in the intervention’s theory of change [12]. When considering telemonitoring, the hard core can be conceptualized as an intervention that leverages technology to enable the collection and transmission of patient biometric data to be viewed and acted upon by a clinician at a distant location [1].

In contrast, the adaptable soft periphery represents the different ways this intervention can be delivered in practice. Adaptability of this soft periphery to local contexts allows innovations to spread without negatively impacting the intervention’s ability to yield desirable outcomes [15], thus maintaining intervention fidelity. As it relates to telemonitoring, elements of the soft periphery may include differences in the hardware used, intensity of clinician monitoring, duration of a telemonitoring program, and format of training or technical support services. However, many of these program components are essential for a telemonitoring program to function. Therefore, delineating the line between the hard core and soft periphery of complex interventions such as telemonitoring programs is particularly difficult. Despite this challenge, implementation and scaling require a clear definition of a program’s core components to ensure that fidelity is maintained when adaptations are undertaken to ensure implementation and scaling success [10,12].

### Sustaining and Scaling a Smartphone-Based Heart Failure Telemonitoring Program

In fall 2016, an HF telemonitoring program was made available to patients of a heart function clinic at an urban hospital in Toronto, Canada. A previous study concluded the initial implementation to be a success based on the degree of integration within the clinic, number of patients enrolled, and fidelity of program delivery as part of the standard of care [16]. However, this study also identified important barriers related to the cost of the equipment and supporting human resources, which could hinder the sustainability and scalability of this program [16]. The objectives of this paper were to (1) understand which components of the telemonitoring program could be modified to reduce costs and adapted to other local contexts while maintaining program fidelity and (2) describe the changes made to the program to enable its sustainability within the initial implementation site and scalability to other health organizations.

## Methods

### Study Design

This qualitative study was designed to elicit insights from end users to better understand the hard core and soft periphery of an existing telemonitoring program. These insights would inform adaptations required to reduce costs of delivering the intervention. Semistructured interviews were conducted within the context of a larger quality improvement program evaluation [17], which was approved by the University Health Network (UHN) Research Ethics Board (16-5789).

### The Existing Heart Failure Telemonitoring Program

#### Integration Within the Standard of Care

The *Medly* program was implemented as part of the standard of care at the UHN Heart Function Clinic in Toronto, which serves patients with complex and advanced HF. Other services currently embedded within the clinic include in-depth teaching from clinic staff about the chronic nature of HF, necessary lifestyle changes, and how to manage complex medication schedules. Typically, relatively stable patients are seen for regular follow-up visits every 6 months, with more acute patients seen more frequently as required. It is also not uncommon for patients to consult with clinic staff over the phone or by email in between visits. The *Medly* program is intended to enhance these existing health services, not replace them.

#### The Medly Telemonitoring System and Services

**Figure 1:**
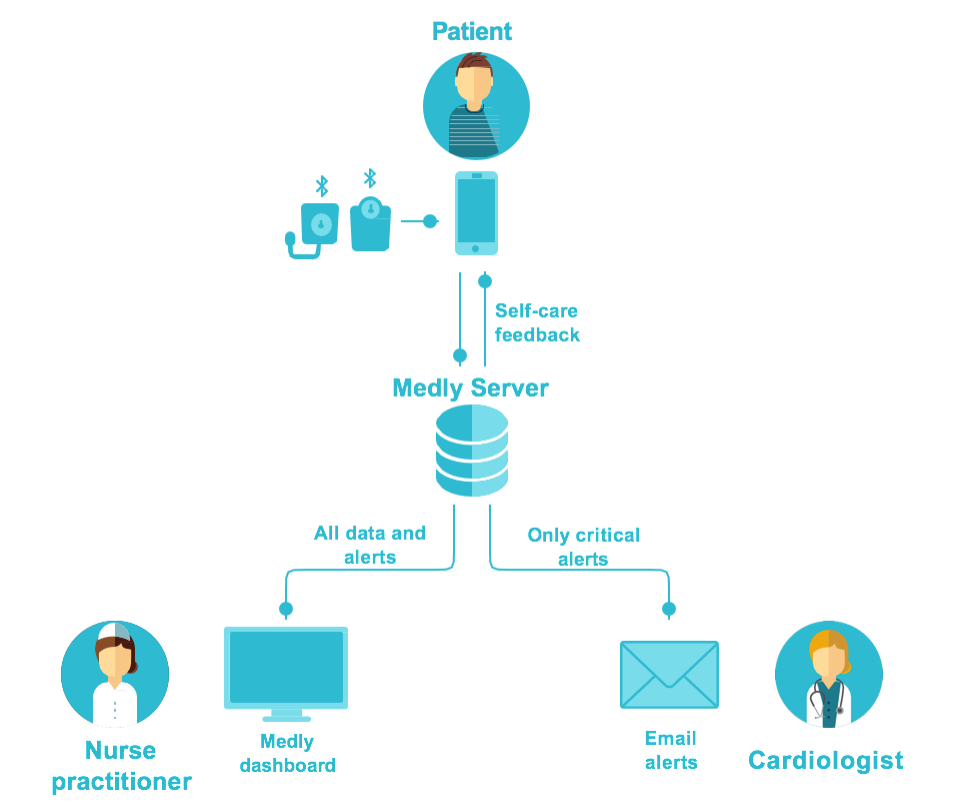
Existing roles and information flows in the *Medly* program.

Central to the *Medly* program is an algorithm-based smartphone app, which patients use to record daily weight, blood pressure, heart rate, and symptoms as soon as they wake up. If there are signs of deterioration in a patient’s health, the *Medly* algorithm triggers a self-care message displayed to the patient in the *Medly* app. In addition, an alert is sent to both a nurse practitioner (NP; via a secure Web-based clinical dashboard) and the most responsible physician (MRP) via automated emails (Figure 1). The MRP is the physician who has overall responsibility for directing the medical care of a patient; in the context of the *Medly* program, this refers to the staff cardiologist responsible for the longitudinal care of patients in the Heart Function clinic. Typically, the NPs are responsible for acting on the alerts during weekdays, with the MRP taking over responsibility for more critical alerts and for responding to all alerts during off hours (evenings and weekends). An earlier version of this intervention was evaluated in a randomized controlled trial of 100 patients that demonstrated improved patient self-care and quality of life compared with a control group [18].

The decision to enroll patients is based on clinicians’ judgment in collaboration with patients. To decide whether someone would be a good candidate, clinicians consider disease severity (usually New York Heart Association [NYHA] classification class 2 or 3), need for self-care support, and a perception that they can adhere to taking daily measurements and be engaged enough to follow self-care instructions provided by the telemonitoring system or the care team. Similarly, the decision to end participation in the *Medly* program is determined jointly between the patient and clinicians. Unlike many other telemonitoring interventions, there is currently no specified end date; patients remain in the program for as long as they are perceived to be benefiting.

**Table 1:** Opportunities for program adaptation probed in the user interviewers.

Program component	Rationale
Peripheral devices	To understand if providing all patients with standardized Bluetooth-enabled peripheral devices free of charge is an essential component of a telemonitoring program. A *bring your own device* (BYOD) model, whereby patients use existing equipment (smartphone, blood pressure cuff, and weight scale), would drastically reduce costs of delivering the program.
Technical support services	One-on-one technical support is resource intensive. Exploring alternative modalities of offering this service could lower direct and opportunity costs by decreasing the time taken to perform these tasks.
Clinician role	Knowing the minimal clinician qualifications for monitoring alerts could save costs because of differences in salary, reimbursement models, and scopes of practice across professions.
Duration of patient enrollment and intensity of monitoring (business hours vs 24/7)	The literature neither provides consistent answers regarding the optimal duration of enrollment nor the intensity of monitoring in a telemonitoring service [8]. Understanding the degree to which these program components can be adapted while maintaining fidelity can produce cost savings through the optimization of resources and inform scaling strategies for telemonitoring programs.

The program was launched by providing patients with a *Medly* kit, which includes a smartphone installed with the *Medly* app, a Bluetooth-enabled weight scale, and a blood pressure cuff, which allows for automatic data transfer from these devices to the *Medly* app. A telehealth analyst (THA) role was created within UHN’s telehealth department to provide technical support by telephone, email, or in person to both patients and clinician user groups. In addition, the THA role included the management of inventory and onsite face-to-face training for each new user. Further details of the program have been published elsewhere [16,17].

### Interview Guide Development

Separate interview guides were developed for patients and clinicians to inform possible strategies for lowering costs and improving program efficiency by gaining a better understanding of the program’s soft periphery. Specifically, participants were asked to comment on the topics presented in Table 1. In their responses, participants were encouraged to consider HF telemonitoring in general, and not just the *Medly* program. The *Medly* software (with embedded rules-based algorithm) was developed around the program’s theory of change [19]. As such, it is considered part of the program’s hard core; thus, no probes related to this component were included.

### Recruitment

Patients (n=23) were identified through purposeful sampling based on age, gender, and time since enrollment in the *Medly* program to ensure a variety of perspectives. This included patients who were interviewed immediately after enrollment and, thus, had no prior experience being monitored in the program. Interviews with patients were conducted until theme saturation was reached (no new themes or perspectives were found in the data) [20]. This was achieved by setting an a priori target of 20 patient interviews. Three additional interviews were conducted, which yielded no new findings, thus confirming theme saturation. All clinicians actively monitoring patients using the *Medly* system at the time of the interviews (n=4) were invited to participate. In addition, 4 clinicians within the UHN Heart Function Clinic who had not yet begun using the system were also interviewed to obtain the views of nonusers. Written informed consent was obtained from all participants.

### Interview Procedures and Analysis

Patients had the option of being interviewed in a private room at the UHN Heart Function Clinic during one of their regularly scheduled visits or over the phone. Clinicians were interviewed in their private offices. Interviews lasted 15 to 60 min and were recorded and transcribed verbatim. Transcripts were analyzed using conventional content analysis [21]; PW and KG each independently coded the transcripts and then met to discuss the results and discrepancies with themes. Once a finalized coding scheme was agreed upon, it was used to code the transcripts before a final analysis of themes. NVivo version 11 (QSR International, Doncaster, Victoria, Australia) was used to organize the data analysis.

### Adapting the Telemonitoring Service

On the basis of the qualitative findings, PW and KG interpreted the degree to which each of the *Medly* program components explored in the interviews could be adapted without impacting program fidelity. Ideas for redesign were discussed during biweekly operations meetings and prioritized for implementation according to their (1) potential to impact sustainability and scalability through cost reductions and optimization of clinic resources, (2) feasibility of implementing the change, and (3) perceived risks of negatively influencing program fidelity (and ultimately effectiveness).

## Results

### Demographics

The demographic characteristics for the patients interviewed were representative of the patients enrolled in the *Medly* program. The average age was 60 years (SD 15) and 74% were male (17/23); see additional demographic characteristics and clinical variables (*NYHA* and left ventricular ejection fraction) in Table 2.

**Table 2:** Characteristics of patient interview participants.

Characteristic		Statistics
Age (years), mean (SD)		60 (15)
**Sex, n (%)**		
	Male	17 (74)
	Female	6 (26)
**Ethnicity, n (%)**		
	White	14 (67)
	Other	7 (33)
**Place of birth, n (%)**		
	Canada	12 (57)
	Other	9 (43)
**Highest education achieved, n (%)**		
	Less than high school	1 (5)
	High school	6 (29)
	College or university	14 (67)
**Rurality, n (%)**		
	Urban	8 (38)
	Suburban	9 (43)
	Rural	4 (19)
**Income in Can $, n (%)**		
	<$15,000	3 (14)
	$15,000-$49,999	8 (38)
	>$50,000	6 (29)
	Preferred not to answer	4 (19)
**Supplementary health insurance, n (%)**		
	Yes	14 (70)
	No	6 (30)
**New York Heart Association classification, n (%)**		
	Class 2	12 (52)
	Class 3	11 (48)
Left ventricular ejection fraction, mean (SD)		33 (13)

At the time of the interviews, 13% (3/23) of patients had been enrolled for 12 months; 48% (11/23) had been enrolled for 6 months; and 9% (2/23) had been enrolled for 1 month. In addition, 22% (5/23) were interviewed immediately after receiving training on their first day and, thus, had no prior experience being monitored in the *Medly* program. Of the 8 clinicians who participated, 2 NPs and 2 cardiologists had 9 to 12 months of experience monitoring patients with the *Medly* system. The remaining 4 cardiologists had no first-hand experience monitoring patients in the *Medly* program.

### Interview Findings

The following is a discussion of participants’ perceptions of opportunities for adapting existing program components aimed at reducing costs and optimizing clinic resources. Themes included were as follows: (1) Bring Your Own Device (BYOD), (2) technical support, (3) clinician role, (4) duration of patient enrollment, and (5) intensity of monitoring.

#### Bring Your Own Device

When the *Medly* program was launched, the intent was to shift to a BYOD model; however, at the time of the interviews, only minimal plans had been made to operationalize this change. The interviews highlight that clinicians were generally supportive of patients using their own equipment as it was necessary to ensure the sustainability of the program:

I think (a BYOD model) is excellent. In fact, I’ve had patients ask me about it...I think, for sure that would be helpful and certainly, more cost effective because we obviously can’t give kits to everybody.

Clinician 3

Some concerns were raised about the questionable quality of patients’ current equipment and the fact that, in the absence of Bluetooth data transfer, patients may accidentally manually enter values incorrectly. The possibility of patients purposefully entering inaccurate information was raised by 4 clinicians, but it was ultimately believed that mutual trust between parties is a prerequisite for any telemonitoring program to be effective:

I guess the only thing you’d have to really make sure of is that they typed things in properly. People make typos, but I guess there would have to be something factored in for a double-check...I don’t believe that people are going to be lying about their numbers. If I thought people were going to lie right, left, and centre, then no, that would be ridiculous and I wouldn’t want to participate in that. But I’d like to believe that if you’re going to commit enough to take the time to take those readings and enter them every day, then I think you’re doing it correctly.

Clinician 3

Although clinicians believed a BYOD model is required for the financial sustainability of the program, they believed some kits need to be available to ensure equitable access to the program. The general opinion was that *Medly* kits should be available for distribution on a case-by-case basis and could be informed by the patients’ socioeconomic status, degree of cognitive impairment, and level of dexterity:

I think there’s still a role for a hybrid kind of model where some people are provided with (the full Medly kit) and some people are provided with the less expensive intervention. You pick your battles and you’d be extremely cautious as to giving a BYOD to someone who has dexterity problems for instance.

Clinician 6

Most patients who received a full *Medly* kit as part of the program said that they would prefer downloading the *Medly* app to their personal smartphone. Common reasons include the inconvenience of being responsible for multiple phones, unfamiliarity with the smartphone provided, and feeling that their health data could be more transportable if it were on their personal device:

I just wish I had more control over it through my [personal] phone because the[n]...I could pull out all those reports from Medly myself and give it to a doctor, a walk-in, anywhere...I was given a phone that I was not familiar with...so it took me awhile to learn it and to get familiarized with it.

HFpro064

One patient who did not own a smartphone said they would prefer if the *Medly* app was available on a tablet:

The only reason why I haven’t bothered getting an iPhone is I have an iPad...I like my iPad because the screen is nice and big...It was never important to me to have a phone that has all the bells and whistles.

HFpro159

A minority of patients interviewed said they preferred having the separate *Medly* phone because they like to keep all the equipment together. Although a separate phone was their preference, all patients said they would use their own device if that was the only option. Many patients understood the economic implications and felt it was reasonable:

I think that it makes it so much easier to have [the phone, weight scale, and blood pressure cuff] all together...I know it might be more cost-effective but it is so much easier on your mental being that you go in, you do what you have to do..[But] you do what you have to do.

HFpro052

A clear majority of patients preferred the convenience of Bluetooth data transfer but also said they would manually enter biometric data if their existing peripheral devices were not Bluetooth-enabled:

The bonus of this whole system is the Bluetooth...Typing in numbers, you [would] get tired of it...For me if I’m looking at my own health, it wouldn’t bother me a bit but I'm different from someone else.

HFpro089

I like the fact that [the data transfer] is done for you. If I had no other choice, then you have no other choice.

HFpro154

Approximately half of the patient participants said they would purchase Bluetooth devices out-of-pocket, but some participants perceived this option as being unfair, echoing clinician concerns of accessibility:

I might [purchase the equipment]...[but] I’m not sure it’s really fair to ask people to do that because you’d automatically filter out a lot people who either couldn’t claim it on insurance or weren’t going to do that...(the) system would all go wrong; it would just be upper middle-class people.

HFpro061

#### Technical Support

Clinicians, not having had direct experience with training and giving technical assistance to patients, did not have strong feelings regarding the format of technical support. However, 1 clinician stated there is an opportunity to minimize resources required for onboarding a patient:

It would be nice as much as possible to automate aspects of the onboarding...because I think actually paying somebody to be there to onboard people will be difficult to scale.

Clinician 1

Another clinician said that although the format of training needs to be appropriate, it is also important that the patient can start with the program immediately after the decision is made as opposed to scheduling training on a future date:

When you go in as a clinician and you have a conversation with the patient about a plan of care and the role of [telemonitoring], what it can offer and why it’s important. You [need] an immediate...“Okay here’s your system, you’ve been immediately trained, you’ve been setup,” versus them going home, 2-3 weeks going by [with the patient thinking] “Oh maybe it’s not that important.”

Clinician 2

All patients described a positive experience with the face-to-face onboarding, but when asked if it was essential, many reflected that it might not be because the system was intuitive to use. Even those who were not tech savvy said they could figure it out at home by themselves or with the help of a family member, especially if they could follow along with a video:

I think the face-to-face was good because I watched [the THA] as she was putting the stuff in and I’m a visual learner...If I see it, it makes perfect sense...I tell people all the time, if you’re stuck on something there’s a video on YouTube of everything...I mean, it was nice having the face-to-face but that’s not always an option.

HFpro159

Although most patients had positive things to say about calling the technical support services, others hesitated before seeking help for fear of being a burden and confusion about who to call:

Well [I didn’t contact technical support because] I just don’t want to bother anybody.

HFpro064_6m

It wasn’t Bluetoothed properly [and] I didn’t really know who to call. I probably had [the] number, but that was kind of a little bit bothersome.

HFpro106_6m

#### Clinician Role

Clinicians believed that the scope of practice of a registered nurse (RN) or NP is well suited for triaging and addressing many telemonitoring alerts. All clinicians agreed that an MRP with HF experience needs to be involved, particularly to deal with the more serious alerts:

I think that the first line of defense is totally appropriate to be nursing with some training in HF because Medly] is a rules-based system and therefore critical alerts should escalate to the physician. The non-critical alerts I absolutely believe that the first line of defense could be a nurse, nurse practitioner, physician assistant, all would be appropriate.

Clinician 1

It’s ultimately a great role for nurse practitioners to champion because you need to have that person that can assess and make a clinical decision about changing a med[ication] or bringing someone in urgently to be seen in the clinic.

Clinician 2

Regardless of the type of professional involved, most respondents believed that telemonitoring programs would be most effective if the clinician receiving and responding to alerts was part of the patient’s care team as opposed to the alerts being sent to a third-party telehealth clinician:

I think one of the issues with Medly...is you still need to have the most responsible person for the Medly involved in the actual patient's clinical care in some way.

Clinician 2

Patients generally agreed with this sentiment, expressing that they prefer the person receiving telemonitoring alerts to have the ability to act immediately. One patient made this point by contrasting the *Medly* program with their previous experience with another telemonitoring program:

I accepted [to be enrolled because they] said [my health information] would go straight to [UHN]...I think [with my previous telemonitoring system] they sent it to [various people] and eventually [my doctor] would see it. But he might be 4th or 5th down the line.

HFpro159

#### Duration of Patient Enrollment

Clinicians felt that patients could eventually be removed from a telemonitoring program if they were no longer actively benefitting (ie, had learned how to self-care or their condition had stabilized). However, a generalizable duration of enrollment could not be established:

Some [patients] just might like the comfort of knowing that [they]’re tied into a clinical team that’s still there if you need help. But I think you have to look at it from your larger team because you can’t just have endless people enrolled in the program, you probably will have to have a maximum at some [point]...I think if someone’s been really stable for 6 months, they haven’t had a lot of alerts, they are very confident as to what their target weight is, what they need to do in terms of lifestyle modifications and symptoms to watch for, then they’ve learned what they needed to learn in that 6 months and they don’t require [the program].

Clinician 2

I think there may be an optimal time to improve self-care...there may be a curve and the curve plateaus and there may not be any further incremental benefit to self-care other than knowing that there is this rules-based system keeping an eye on them right. So it may be that you optimize self-care within 3 months...but patients [might] want to stay on it. And again, if you can really demonstrate value I don’t have a problem with that.

Clinician 1

Many patients spoke of HF as being a lifelong condition and that they would like to stay in the program for as long as possible or until something came up that made the program unnecessary, such as undergoing a heart transplant:

I think for me [HF is] a lifestyle thing now. I think I’d be a fool not to use [Medly], I guess that sums it up. I was so sick and dead that I take my recovery very seriously...I think I’d be a fool not to take advantage of it.

HFpro107

I’ve got a lifelong condition so I don’t really see an end time, unless I end up going for a heart transplant, which I’m not going to hopefully have to do anytime soon. So yeah, I think [my participation] will be ongoing.

HFpro019

#### Intensity of Monitoring

Clinicians recognized that asking clinicians to be available at all times to receive and respond to telemonitoring alerts is not scalable. However, they also strongly felt that these interventions are most effective if there is someone monitoring alerts 7 days per week:

It’s not really fair for a single person to be on-call 24/7. You have to take that into consideration in terms of physician burnout and all those things. There should be a mechanism to deal with that, whether it goes to the physician on-call or something like that. But I do think that in order for this to be effective, a 24/7 tool would be more appropriate than a business hours tool because it’s not like people get sick only during business hours.

Clinician 6

Although participating clinicians said they would strive to have alerts monitored 7 days per week, their responses also highlighted that the requirements for intensity of monitoring are dependent on the telemonitoring system itself. For example, many clinicians highlighted that the rules-based algorithm in the *Medly* system provides patients with clinically validated messages, allowing for a form of 24/7 feedback even if a clinician is not always available:

I may be camping somewhere where I am not accessible. But I think the whole thing of the Medly system is it doesn’t rely on me [seeing] the alerts, the patients are instructed to do things [by the algorithm]. We have set up a plan and they have to act accordingly. They don't have to wait for me to respond to [follow the instructions].

Clinician 8

Patients both with and without experience in the *Medly* program felt that someone should be available to respond to alerts 7 days per week but that this may also be contingent on the disease severity of the patients enrolled in the program:

If somebody weren’t that sick and they just had a bit of a heart issue, I don’t know if they would like this big brother, big sisterly thing where the [clinicians] call first thing in the morning on Sunday...I love that part. I think that’s the essence of the system...I mean [my doctor] is a world-renowned cardiologist and she calls me on Sunday morning at 7, because my reading is a little high. I can’t believe it, it’s the ultimate professionalism. If she didn’t, I wouldn’t be heartbroken, but I just think that she uses the system as it should be used.

HFpro107

### Redesign of the Medly Program for Sustainability and Scalability

**Figure 2:**
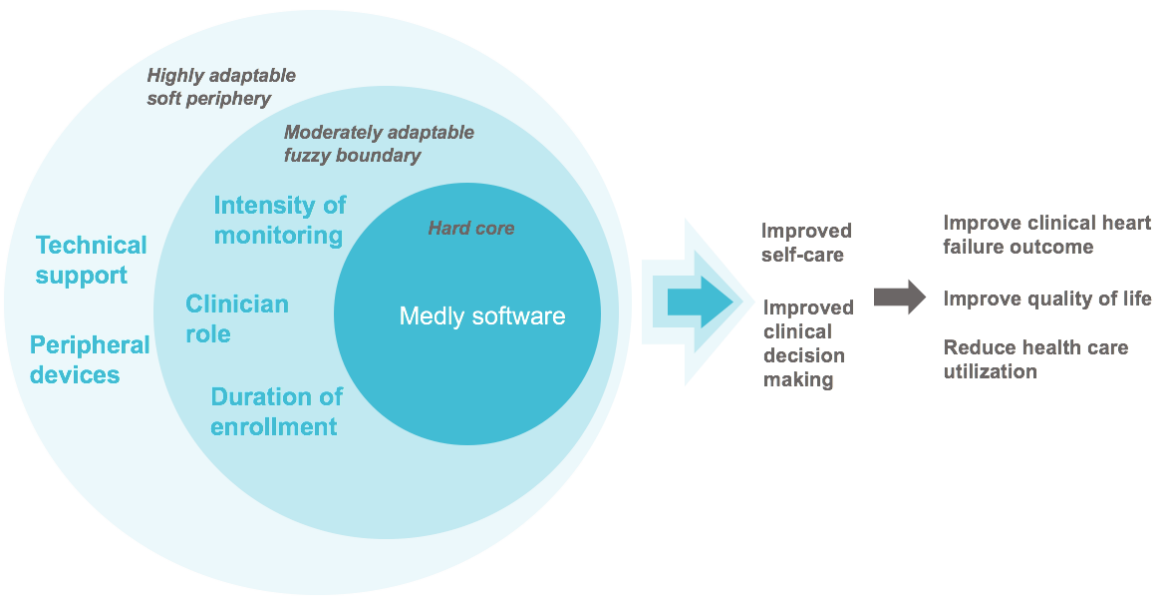
Hard core and soft periphery of the Medly program as informed by user interviews and its role in the intervention’s theory of change.

Qualitative results related to opportunities to modify components of the *Medly* program were interpreted and classified according to the degree to which they could be adapted while maintaining program fidelity. As shown in Figure 2, the format of technical support and the peripheral equipment used were considered highly adaptable and, thus, clearly part of the *Medly* program’s soft periphery. The participation of a clinician (role and intensity of monitoring) and the monitoring of patients over time are central components of any telemonitoring program theory of change, indicating some overlap with the intervention’s hard core. However, the interviews suggest some degree of adaptability depending on contextual factors, which explains why intensity of monitoring, clinician role, and duration of enrollment were classified as moderately adaptable and part of a *fuzzy boundary* between the hard core and soft periphery. These findings informed the decisions to adapt the *Medly* program as described in Table 3.

**Table 3:** Adaptations to the *Medly* program to ensure sustainability and scalability.

Opportunities for adaptation	Decisions related to the *Medly* program
Peripheral devices	Move forward with the implementation of a hybrid Bring Your Own Device model, whereby most patients use their own mobile and peripheral devices with some *Medly* kits still being distributed to patients in need (eg, lack of ability to pay, low cognitive ability, and dexterity problems). This involved change to the operational procedures, including (1) generation of a list of recommended clinically valid weight scales and blood pressure cuffs for patient purchase, (2) clinician prescription of peripherals so that costs can be reimbursed by private medical insurance or tax deductions, and (3) expanding technical troubleshooting procedures to cover the most common devices on the market. Software development needed to implement this decision included (1) the development of a manual entry version of the *Medly* app with features to protect against inaccurate data entry and (2) adapting the *Medly* app for tablets. The one-time costs of this developmental work are being incurred by the organization developing the *Medly* system. Thus, it is not considered part of the program’s implementation costs.
Technical support	A website was built containing patient training content and an extensive frequently asked questions section. It is expected that this website will allow patients to be more self-sufficient and greatly reduce the number of calls made for technical support. In addition, development is underway to build a self-training feature directly into the *Medly* app. This will also increase the feasibility of providing same-day onboarding by minimizing scheduling challenges that exist with face-to-face training. The shift toward lower-touch technical support made it possible for most of the frontline technical support tasks (patient training, managing inventory, and basic troubleshooting) to be taken up by clinic staff. It is believed that this model more closely resembles what will be feasible in most health care settings, and it is expected to improve the patient experience by having a single point of contact.
Clinician role	An RN^a^ was hired to take over the primary clinical management of alerts from the existing nurse practitioners as well as the technical support role from the existing telehealth analyst. This RN was responsible for triaging alerts and escalating clinical issues to MRPs^b^ when necessary.
Duration of patient enrollment	No change. A universally applicable duration of enrollment could not be determined as it depends on patient characteristics.
Intensity of monitoring	No change. The 7 days/week monitoring will be maintained at the HF clinic with cardiologists volunteering their time to cover weekend alerts and transferring alerts to a colleague if they will be unavailable for extended periods. Modifications are being made to the *Medly* dashboard to facilitate the transfer of alerts from one MRP to another.

^a^RN: registered nurse.

^b^MRP: most responsible physician.

## Discussion

### Principal Findings

This qualitative study is the first to describe adaptations to an existing HF telemonitoring program aimed at enabling its sustainability and scalability. The redesign was informed by interviews with clinicians and patients to identify which program components could be adapted while maintaining program fidelity. User perceptions helped identify that the type of peripheral devices used and the format of technical support were highly adaptable, making them ideal targets for cost reduction measures. This led to the decision to move forward with a hybrid BYOD model and lower-touch technical support services, which would substantially reduce the cost burden to the clinic for delivering the program. In addition, findings related to the clinician role confirmed that frontline alert management should be done by someone within the patient’s immediate circle of care rather than being outsourced to an offsite telehealth clinician. This informed a more cost-effective model in using an RN to replace the existing NPs as the frontline manager of telemonitoring alerts and to absorb the technical support functions previously performed by the THA. The notion of an RN playing both the central clinical and operational roles within telemonitoring services is supported in the literature [22,23]. In this case, hiring of an RN made sense as a resource optimization measure because of the existing program structure, which involves escalating alerts to an MRP. In sites where a physician is not as readily available, a professional with an ability to make medication changes (eg, NP) might be more appropriate to lead a telemonitoring service.

Because no generalizable dose with respect to duration of enrollment and intensity of monitoring could be established, no changes were made at the existing program site. However, the moderately adaptable nature of these components may reveal opportunities for scaling as they might be tailored to allow for program integration within sites with different patient populations, resources, and objectives. For example, although rapid feedback from a clinician is often described as the most important component of a telemonitoring service [24], a clinical site serving patients with a lower disease severity may not require 7 days per week monitoring. In addition, a site with resource constraints may wish to prioritize improving patient self-care, in which case, a 3- to 6-month duration might represent an optimized duration of enrollment. Alternatively, sites with available resources and different organizational values may wish to prioritize the patient’s experience in addition to improving self-care and decide to monitor patients indefinitely. Finally, although the clinicians in this study felt comfortable receiving alerts during off hours, the medicolegal implications of continuous monitoring must be considered on a site-by-site basis. For example, it is possible that 7 days per week monitoring is deemed important for a specific patient population but that receiving alerts during off hours represents a medicolegal concern that cannot be addressed through the hiring of additional staff or on-call personnel. Such a situation may leave a site no choice but to offer a weekday-only telemonitoring program, with the understanding that the impacts of the program may be suboptimal.

### Comparison With Prior Work

Several studies have explored the barriers to and facilitators of implementing telehealth systems [25,26] but few have described the process of adapting an existing program to ensure its sustainability and scalability. One multiple case study by Taylor et al describes a participatory approach to implementing solutions for expanding a telehealth program [27], but the description of these activities remained high level without concrete examples, leading to limited transferability of results.

Many authors cite the ubiquity of smartphones as an opportunity for delivering telemonitoring services at a lower cost [28,29]. However, most studies of mobile phone–based telemonitoring have provided patients with this mobile equipment [30], and little is known about clinicians’ and patients’ perceptions of a BYOD model. From a usability perspective, there is a clear preference among patients, both in the literature [24,31,32] and in this study, for using Bluetooth-enabled peripheral devices. However, what appears most important is that patients can access telemonitoring services using devices they are most familiar with (ie, personal smartphones and tablets) [33-35] and that the perceived advantages of a telemonitoring program are greater than any usability inconveniences caused by manually entering biometric data [36-38]. To our knowledge, ours is the first study to confirm that BYOD is perceived by both clinician and patient users as a viable option for delivering telemonitoring services with a caveat that considerations are required to ensure universal accessibility.

The finding that clinicians believe patients could exit a telemonitoring program after they have stabilized or gained self-care skills is supported by other studies [39]. We found a similar perspective among clinicians in this study, but we also found that many patients grow accustomed to being remotely monitored and would like to continue over a longer term. Until now, considerations about the duration of telemonitoring interventions have primarily been driven by costs. However, this perspective ignores the natural history of HF, whereby although patients may stabilize for a period, they will rarely improve [40]. Therefore, as opportunities are leveraged to deliver telemonitoring interventions at lower costs (including clinicians’ time through the development of more sophisticated decision-support capabilities), it is conceivable that the costs of delivering certain telemonitoring programs will become sufficiently low so that it removes the need to ration their use. Thus, future work should seek to answer whether it is better (from the patient, clinician, and health system perspectives) to (1) remove patients from a low-cost telemonitoring program when they have stabilized only to reinstate them in the program when their condition has worsened or (2) leave them enrolled in the program indefinitely.

### Limitations

First, although participants were asked to consider their responses with respect to telemonitoring in general, it is likely that their responses were influenced by their experiences with the *Medly* program. In stating this limitation, we emphasize that our intent was to describe adaptations to a specific telemonitoring program rather than to provide a detailed blueprint for implementing all HF telemonitoring interventions in any given clinical context. We argue that the context-dependent nature of implementing complex interventions makes the creation of such a blueprint impossible. Rather, we have sought to provide foundational considerations for developers of telemonitoring programs and for implementation scientists who wish to sustain and scale existing telemonitoring programs to other clinical sites and health care organizations. Second, the pragmatic nature of this study meant that patients can be enrolled in the *Medly* program without consenting to participate in the evaluation activities, making them ineligible to participate in the interviews. Our inability to purposely sample these patients may have led to selection bias. Third, the interview guides were developed to probe the opinions of users on specific program components. We recognize that our approach for compartmentalizing and defining the various components of this complex intervention was subjective and context specific; this should be considered when determining the transferability of results to alternative settings. Finally, although the resulting user-guided adaptations are expected to maintain the fidelity of the intervention, the true impact of these changes was not empirically tested in this study. This important question will be evaluated as part of a subsequent publication on the overall impacts of the *Medly* program as well as patient adoption and adherence to the intervention.

### Conclusions

Theories of diffusion of innovation suggest that one of the keys to scaling health interventions lies in adapting elements of its delivery to better fit the implementation context. However, this is only true if fidelity of the intervention can be maintained and its potential effectiveness is not compromised. This concept has informed the implementation of cost reduction measures of an existing HF telemonitoring program to ensure its sustainability. Our findings suggest that the peripheral devices used in telemonitoring programs and the format of technical support are highly adaptable, making them ideal targets for cost reduction measures. Duration of enrollment and intensity of monitoring are inextricable components of a telemonitoring intervention, but the dose of these components required to yield expected outcomes is highly context dependent. Our efforts provide a user-centered example of how necessary actions can be taken to improve the sustainability and scalability of telemonitoring programs.

## Abbreviations

BYOD: bring your own device
HF: heart failure
MRP: most responsible physician
NP: nurse practitioner
NYHA: New York Heart Association
RN: registered nurse
THA: telehealth analyst
UHN: University Health Network
